# Supplementary material for: Content-rich biological network constructed by mining PubMed abstracts
Source: BMC Bioinformatics. 2004 Oct 8;5:147. doi: 10.1186/1471-2105-5-147 (PMC528731; doi:10.1186/1471-2105-5-147)
Supplement: Additional File 5 — The original Chilibot query results of the term "long-term potentiation (LTP)" and 22 other terms, limiting the latest references analyzed to the years 1990, 1995, 2000, and 2004. [file 1471-2105-5-147-S5.bz2 › chilibotAdditionalFile5/ltp2004/html/SYNAPSIN I_TRKB.html]

 


 **SYNAPSIN I** and **TRKB** 
  
Found 6 abstracts in PubMed,  **6 abstracts were retrieved and analyzed**.  


---

 Search Google  |
 PDF files only 
|  EDU domain only 

---

**Interactive relationship** (e.g. stimulation, inhibition, etc)

**Parallel relationship** (e.g. studied together, co-existance, homology, etc.)

- Brain derived neurotrophic factor BDNF, its signal transduction receptor  **trkB** , and its downstream effector,  **synapsin I** , were measured in the hippocampus and occipital cortex of young animals after fluid percussion brain injury FPI.  Ref: 12184851 J Neurotrauma, 2002
- **Synapsin I** , a synaptic mediator for the action of BDNF on neurotransmitter release, increased in proportion to GAP 43 and  **trkB**  mRNA levels.  Ref: 12424260 J Neurophysiol, 2002
- At postinjury day 7, both  **trkB**  and  **synapsin I**  mRNA expression increased ipsilaterally and decreased contralaterally in the occipital cortex.  Ref: 12184851 J Neurotrauma, 2002
- The selectivity of the changes in  **trkB**  and  **synapsin I** , affecting the hippocampus, is in agreement with the role of this structure in processing of spatial information.  Ref: 11516407 Brain Res, 2001
- Therefore, parallel changes in  **TrkB**  and  **synapsin I**  mRNAs suggest a role of the BDNF system in synaptic function or adaptation.  Ref: 11516407 Brain Res, 2001
- Levels of  **TrkB**  mRNA in the hippocampus were attenuated after learning acquisition Day 20, but  **synapsin I**  mRNA was still elevated, suggesting that the BDNF system may participate in events secondary to learning, such as strengthening of neural circuits.  Ref: 11516407 Brain Res, 2001
- Nuclease protection assays showed maximal levels of  **TrkB**  and  **synapsin I**  mRNAs in the hippocampus by the time that asymptotic learning performance had been reached Day 6.  Ref: 11516407 Brain Res, 2001
- **TrkB**  and  **synapsin I**  mRNAs showed an increasing trend in the cerebellum of learning rats and no changes were observed in the caudal cerebral cortex.  Ref: 11516407 Brain Res, 2001
- In fact, BDNF potentiates intracellular calcium release presynaptically and BDNF induced  **TrkB**  activation also results in phosphorylation of  **synapsin I**  via mitogen activated protein kinase, which increases the number of synaptic vesicles available for release.  Ref: 14695476 Sheng Li Xue Bao, 2003
- The use of a novel microbead injection method in our blocking experiments and Taqman reverse transcription polymerase reaction RT PCR for RNA quantification, have enabled us to evaluate the contribution of different pathways to the exercise induced increases in the mRNA levels of BDNF,  **TrkB** , CREB, and  **synapsin I** .  Ref: 14622908 Neuroscience, 2003
